# Short-Term Effects of Lupin vs. Whey Supplementation on Glucose and Insulin Responses to a Standardized Meal in a Randomized Cross-Over Trial

**DOI:** 10.3389/fphys.2017.00198

**Published:** 2017-04-10

**Authors:** Kathrin Schopen, Ann C. Ewald, Bernd W. Johannes, Wilhelm Bloch, Jörn Rittweger, Petra Frings-Meuthen

**Affiliations:** ^1^Department of Space Physiology, Institute of Aerospace Medicine, German Aerospace CenterCologne, Germany; ^2^Department of Molecular and Cellular Sport Medicine, Institute of Cardiovascular Research and Sport Medicine, German Sport University CologneCologne, Germany; ^3^Department of Pediatrics and Adolescent Medicine, University of CologneCologne, Germany

**Keywords:** *Lupinus albus*, lupin, whey, blood glucose, serum insulin, dietary protein, postprandial responses, glycemic control, The trial was retrospectively registered on ClinicalTrials.gov under number NCT02413671.

## Abstract

**Background:** Whey protein is known to reduce postprandial glycaemia in people with type 2 diabetes mellitus. Lupin as a vegetable source of protein could be considered as an alternative, as the percentage of vegetarian and vegan consumers is raising. The present study compares the acute glycemic effects of whey and lupin in healthy volunteers following a carbohydrate-rich reference meal.

**Methods** In cross-over design, three standardized meals (reference meal; reference meal + whey; reference meal + lupin) were provided to 12 healthy male and female volunteers, aged between 23 and 33, in a balanced, randomized order. Volunteers' blood glucose and insulin concentrations were analyzed at baseline and at seven time points following the ingestion of the meals.

**Results:** The supplementation of whey or lupin significantly blunted the postprandial increase in blood glucose concentrations compared to the reference meal (*p* < 0.001). In the overall statistical analysis, this effect was comparable for whey and lupin [Δ AUC whey-lupin = 8%, 0–60 min area under the curve (0–60 min AUC), *p* = 0.937], with a blunting effect of −46% by whey (*p* = 0.005, 0–60 min AUC) and of −54% by lupin (*p* < 0.001, 0–60 min AUC). When comparing whey and lupin data only, the insulin increase was found to be more pronounced for whey protein than for lupin supplementation (Δ AUC whey-lupin = 39%, 0–60 min AUC, *p* = 0.022). However, when comparing the insulin response of each supplementation to the one of the reference meal, no differences could be detected (whey *p* = 0.259, 0–60 min AUC; lupin p = 0.275, 0–60 min AUC).

**Conclusions:** Results suggest that lupin and whey can both lower the increase of postprandial blood glucose concentrations to a comparable extent, implying the usability of lupin to reduce postprandial glycaemia. However, the insulin response following the supplementations to a carbohydrate-rich meal seems to differ for these two protein sources.

## Introduction

The development of insulin resistance and high blood glucose concentrations in people with type 2 diabetes mellitus (T2DM) are often linked to obesity, physical inactivity or poor diet (International Diabetes Federation, 2015). Notably, these manifestations can be prevented, or even reversed by physical exercise and dietary interventions (International Diabetes Federation, 2015). Such dietary interventions may include increasing the consumption of food items with a low glycemic index (GI) (Brand-Miller et al., 2002) or a low glycemic load (GL) (Dong et al., 2011), i.e., numbers which indicate a food's potential of increasing postprandial blood glucose concentrations after consumption. Furthermore, food can be supplemented with certain proteins (Gannon et al., 1988, 2003; Nilsson et al., 2004; Frid et al., 2005; Pal et al., 2010; Bertoglio et al., 2011; Dove et al., 2011; Baldeón et al., 2012; Fornasini et al., 2012; Silva Ton et al., 2014).

Whey protein has come into focus over the last decade for its benefits in T2DM. The protein is a mixture of globular proteins isolated from whey, which is a by-product of cheese production and known to stimulate insulin release and to lower postprandial glycemia in type 2 diabetic patients (Frid et al., 2005) as well as in healthy participants (Nilsson et al., 2004). Nevertheless, as an animal protein with a high content of sulfur-containing amino acids, it might lead to so called low-grade metabolic acidosis if taken in high amounts (Frassetto et al., 2001). Changing the body's acid-base homeostasis toward the acidic side might entail a number of undesirable effects, such as an increased bone resorption (Frings-Meuthen et al., 2008), or the stimulation of muscle protein degradation, either by affecting the regulation of branched-chain amino acid metabolism (May et al., 1987) or by activating the adenosine triphosphate-dependent pathway (Mitch et al., 1994). Moreover, evidence relates the risk of hip fracture to high animal protein intake (Frassetto et al., 2000). Taken together, this forms a rationale to seek for alternative ways of protein supplementation, most intuitively by protein from vegetable sources.

The question therefore arises whether such vegetable proteins have similar effects upon glucose metabolism as whey protein. Because of its lower content of sulfur containing amino acids such as cysteine and methionine, we have become especially interested in lupin seeds. The lupin flower belongs to the legume family (Fabaceae) and produces seeds that contain a high amount of protein (up to 40%). Very interestingly, it has been shown to reduce postprandial hyperglycemia (Bertoglio et al., 2011; Dove et al., 2011; Baldeón et al., 2012). Additionally, it could serve as vegan alternative to whey protein, as the percentage of vegetarian and vegan consumers is increasing rapidly over the last years. Nevertheless, there is no direct comparison yet between whey and lupin's effects upon glycemic control.

Hence, the present study was performed in order to compare the effect of whey and lupin supplementation to a carbohydrate-rich reference meal on postprandial blood glucose and insulin responses in healthy human participants. We hypothesized that lupin would lower postprandial glycemia to a comparable extent as whey.

## Participants and methods

### Participants and study design

Five healthy male (height: 1.82 ± 0.08 m, body weight (BW): 81.8 ± 13.16 kg, body mass index (BMI): 24.72 ± 2.3 kg/m^2^, age: 28 ± 3.67 y) and seven female volunteers (height: 1.69 ± 0.08 m, BW: 69.69 ± 5.52 kg, BMI: 20.92 ± 1.63 kg/m^2^, age: 26.86 ± 3.44y) participated in this cross-over designed study, which consisted of three test visits (Table 1). All participants were recruited in-house by word-of-mouth (Supplementary Material, Image 1). An *a priori* sample size estimation was performed with GPower (version 3.1.9.2, university of Kiel, Germany). F-test based MANOVA with an effect size of 0.57, a *p*-value of 0.05 and a power of 0.80 yielded a required sample size.

**Table 1:** **Participants characteristics (n=12, individual values)**.

**Subject**	**Gender**	**Height (m)**	**BW (kg)**	**BMI (kg/m^2^)**	**Age (y)**
A	f	1.57	52.50	21.30	27
B	f	1.71	66.00	22.57	31
C	f	1.84	67.30	19.88	32
D	m	1.74	75.00	24.77	25
E	f	1.69	56.00	19.61	25
G	f	1.70	57.00	19.72	23
H	f	1.70	57.00	19.72	24
K	m	1.72	70.00	23.66	24
L	f	1.62	62.00	23.62	26
M	m	1.87	88.00	25.17	30
O	m	1.85	74.00	21.74	28
P	m	1.90	102.00	28.25	33

**Figure 1:**
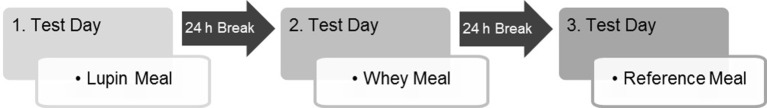
**Exemplary testing protocol for one subject**.

A medical screening was performed before including subjects. Inclusion criteria included fasting plasma glucose levels within the normal range (70–99 mg/dl) and HbA1c within the range of 4–6 %. Participants were single-blinded, and the different test meals were provided in balanced randomized order, with a minimum of 24 h between successive test days. The study lead assigned a number to each of the three test meals and all possible permutations were equally distributed among participants, resulting in two randomly assigned participants receiving test meals in the same sequential order. For the duration of the study, volunteers were asked refrain from high fatty food items (e.g., pizza, fries, etc.), and alcohol. After a fasting blood draw for baseline data (BL1), participants received a standardized breakfast, consisting of rye bread, butter, jam, cheese, ham and either coffee or tea. This was done in an effort to standardize the dietary conditions prior to the actual test meal. The items chosen by participants for breakfast at the first visit were noted, and the same items were presented to each subject for the subsequent two breakfasts. Four hours after consumption of the breakfast, a second blood sample was obtained (BL2), followed by the ingestion of a standardized test meal at lunch time. The latter was tailored to body weight (Table 2) and rich in carbohydrates. It was either provided alone (here called reference meal), or supplemented with either whey (henceforth called whey meal) or with lupin (henceforth called lupin meal) (Figure 1). As in previous studies, a 12-min period had been chosen for meal consumption since glucose and insulin responses are expected to start shortly after meal consumption (Frid et al., 2005). The participants ate steadily over a 12-min time period and finished the complete meals under supervision. Once the meal was finished, further blood samples were drawn at +10, +20, +30, +40, +60, +120, and +180 min through an in-dwelling venous catheter.

**Table 2:** **Nutritional values for the three meals (reference meal, whey meal, lupin meal) per kg body weight**.

	**kJ**	**P (g)**	**F (g)**	**SFA (g)**	**CH (g)**	**S (g)**	**Na (mg)**	**Ca (mg)**	**H_2_O (g)**
Reference Meal	29.1	0.32	0.21	0.06	0.89	0.12	22.6	0.99	4.45
Lupin Meal	38.9	0.69	0.30	0.07	0.90	0.13	22.6	0.99	4.45
Whey Meal	35.9	0.69	0.21	0.06	0.90	0.13	23.9	2.65	4.45

*The table includes calories (kJ), protein (P), fat (F), saturated fatty acids (SFA), carbohydrates (CH), sugar (S), sodium (Na), calcium (Ca) and Water (H_2_O)*.

The study protocol was in accordance with the Declaration of Helsinki, and had been approved by the ethics committee of the Aerztekammer Nordrhein (Ethikkommission der Aerztekammer Nordrhein, Duesseldorf, Germany) before study commencement. The study is registered on http://www.clinicaltrials.gov under trial number: NCT02413671. Written informed consent was obtained from all participants.

### Lunch meals

The reference meal consisted of spaghetti with meat sauce (Apetito, Rheine, Germany) and was supplemented with either lupin or whey or served without supplementation. The amount of supplement was dependent on participants' body weight (Table 2).

### Protein supplementation

Whey protein isolate was obtained as Diaprotein from Dr. Steudle (Linden, Germany) and lupin as sweet lupin flour from BIOTICANA GmbH (Rendswühren, Germany). On the basis of the actual literature, whey protein was supplemented as 0.42 g whey protein per kg body weight. To add an equivalent amount of protein from lupin flour, the reference meal was supplemented with 0.94 g of lupin flour per kg body weight. The respective supplementations were added to and mixed with the sauce and noodles immediately before the meals were served. Nutritional breakdown of sweet lupin flour and whey protein isolate is provided in Table 3.

**Table 3:** **Nutritional breakdown of sweet lupin flour and whey protein isolate per 100 g**.

	**kJ**	**P (g)**	**F (g)**	**SFA (g)**	**CH (g)**	**S (g)**
Lupin Protein	1,047	39.3	9.8	1.5	1.2	1.2
Whey Protein	1,549	90.0	1.2	1.0	2.5	2.5

*The table includes calories (kJ), protein (P), fat (F), saturated fatty acids (SFA), carbohydrates (CH) and sugar (S)*.

The concentrations of methionine and cysteine for both supplementations were analyzed by a local laboratory (Eurofins Laborservice GmbH, Cologne, Germany) using ion-exchange chromatography.

### Blood sample analysis

Blood samples were centrifuged (3000 rpm, 4°C, 10 min) after coagulation. Plasma glucose concentrations were analyzed with an automated analyzer (INTEGRA 400+, Roche, Basel, Switzerland). The remaining plasma was distributed in small tubes and immediately frozen at −80°C until analysis for insulin concentrations.

Insulin concentrations were analyzed with a commercially available ELISA (MERCODIA, Uppsala, Sweden) in the in-house laboratory of the Institute of Aerospace Medicine, Germany with an intra-assay variation of 1.2%.

### Data processing and statistical analysis

The areas under the curve (AUCs) for the glucose and insulin tracings were calculated for each subject and each meal using GraphPad Prism (version 5.01; GraphPad Software Inc, San Diego, USA). All AUCs below baseline were excluded from the calculations. The AUCs were expressed as means ±SDs. Time point analysis was performed to evaluate differences between whey and lupin.

All statistical analyses were performed with IBM SPSS (V.20, Chicago, Illinois). Group mean values with their SEM were calculated for each condition of interest.

For AUC values, a general linear model (GLM) was used to assess significance. For time point analysis a linear mixed-effects (LME) model was used with substance, time and their interaction as fixed effects and participants as a random effect. The respective model means and standard errors are presented in the graphs. An alpha level of 0.05 was set for all tests for significance. Moreover, normal distribution of the raw data was verified by the Kolmogorov-Smirnov-test. The residuals were tested to be normally distributed to confirm the appropriateness of the statistical modeling.

## Results

### Sulfur amino acid content of whey and lupin

Whey protein was found to contain an amount of 2.28 g of methionine and of 2.41 g of cysteine per 100 g. For lupin the content of methionine amounted to 0.13 g, and for cysteine to 0.61 g per 100 g.

### Postprandial response

All meals led to a significant transient increase in postprandial blood glucose (*p* < 0.001) and serum insulin concentrations (*p* = 0.001). The fasting blood glucose and serum insulin concentrations (BL1) as well as the baselines measured directly before the ingestion of the meal (BL2) were comparable between the three test days (blood glucose: all: *p* > 0.69; serum insulin: all: *p* > 0.25).

### AUC analysis

Postprandial blood glucose concentrations (as AUC) are shown in Table 4. Statistically, no differences in glucose concentration were observed between the supplementation with whey and lupin (0–60 min AUC: *p* = 0.937; 0–180 min AUC: *p* = 1.000). Compared to the reference meal, the blood glucose response within the first 60 min after meal consumption was reduced by 45.97 ± 10% (*p* = 0.005) when supplemented with whey protein. Over the interval of 0–180 min, blood glucose AUC was reduced by −34.54 ± 10% for the whey meal (*p* = 0.040). For the supplementation with lupin the 0–60 min blood glucose AUC was reduced by −53.49 ± 9% (*p* < 0.001) for the 0–60 min AUC, and by and −34.64 ± 11% over the 0–180 min interval (*p* = 0.030) when compared to the reference meal.

**Table 4:** **Postprandial area under the curve (AUCs, ± SEM) for blood glucose and serum insulin after the reference, whey and lupin meal in healthy human participants**.

	**Reference**	**Whey**	**Δ (Ref-Whey) [%]**	**Lupin**	**Δ (Ref-Lupin) [%]**	**Δ (Whey-Lupin) [%]**
**GLUCOSE AUC [MG·MIN/DL]**						
0–60 min	1984 ± 174	1072 ± 135	−46 ± 10^**^	923 ± 128	−54 ± 9^***^	8 ± 10
0–180 min	3809 ± 474	2493 ± 342	−35 ± 10^*^	2489 ± 391	−35 ± 11^*^	0 ± 12
**INSULIN AUC [MU·MIN/L]**						
0–60 min	1583 ± 202	1951 ± 257	23 ± 14	1323 ± 186	−16 ± 13	39 ± 15^*^
0–180 min	3216 ± 582	3684 ± 539	15 ± 14	3373 ± 643	5 ± 15	10 ± 15

*** p < 0.001;

** p = 0.001–0.01;

* *p > 0.01–0.05*.

The postprandial serum insulin concentrations (as AUC) are also shown in Table 4.

The insulin response in the 0–60 min AUC was higher following the consumption of the whey meal than after the lupin meal (*p* = 0.022). However, there was no difference found at 180 min after meal ingestion (*p* = 1.000).When compared with the reference meal, neither had whey protein supplementation any detectable effect (0–60 min AUC: +23.20 ± 14%, *p* = 0.259; 0–180 min AUC: 14.54 ± 14%, *p* = 0.656), nor had the supplementation with lupin (0-60 min AUC: −16.42 ± 13%, *p* = 0.275; 0–180 min AUC: 4.78 ± 15%, *p* = 1.000).

### Time point analysis

**Figure 2:**
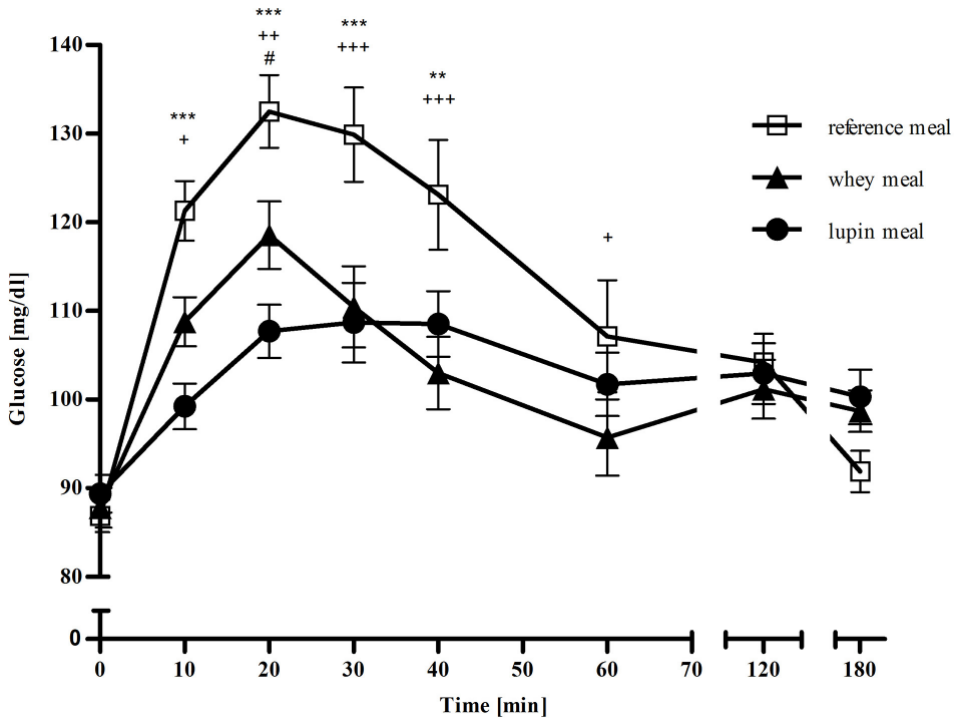
Mean (± SEM) of blood glucose concentrations in mg/dl over 180 min following the three different meals: □ representing the reference meal, ▴ representing the meal supplemented with whey, • representing the meal supplemented with lupin, ^*^ representing differences between the lupin and the reference meal, + representing differences between the whey and the reference meal and # representing differences between the lupin and the whey meal. ^***/+++/###^*p* < 0.001; ^**/++/##^*p* = 0.001–0.01; ^*/+/#^*p* > 0.01–0.05.

Postprandial blood glucose concentrations are shown in Figure 2. Notably, fasting blood glucose concentrations were within the normal range (reference: 86.88 mg/dl; whey: 87.79 mg/dl; 89.39 mg/dl). A significant difference between whey and lupin was observed only at time point +20 min, with lower blood glucose concentrations for the lupin meal as compared to the whey meal (*p* = 0.040). Postprandial blood glucose concentrations were lower at 10 min (*p* = 0.013), at 20 min (*p* = 0.005), at 30 min (*p* < 0.001), at 40 min (*p* < 0.001), and at 60 min (*p* = 0.028) after consumption of the whey meal as compared to the reference meal, but no difference were observed at +120 min and +180 min *p* = 1.000 and *p* = 0.356, respectively). For the supplementation with lupin, with respect to the reference meal, significantly lower postprandial glucose concentrations were observed at 10 min (*p* < 0.001), 20 min (*p* < 0.001), 30 min (*p* < 0.001) and 40 min (*p* = 0.003), whilst no differences were observed for the time points +60 min, +120 min and +180 min (*p* = 0.647, *p* = 1.000, and *p* = 0.163, respectively).

**Figure 3:**
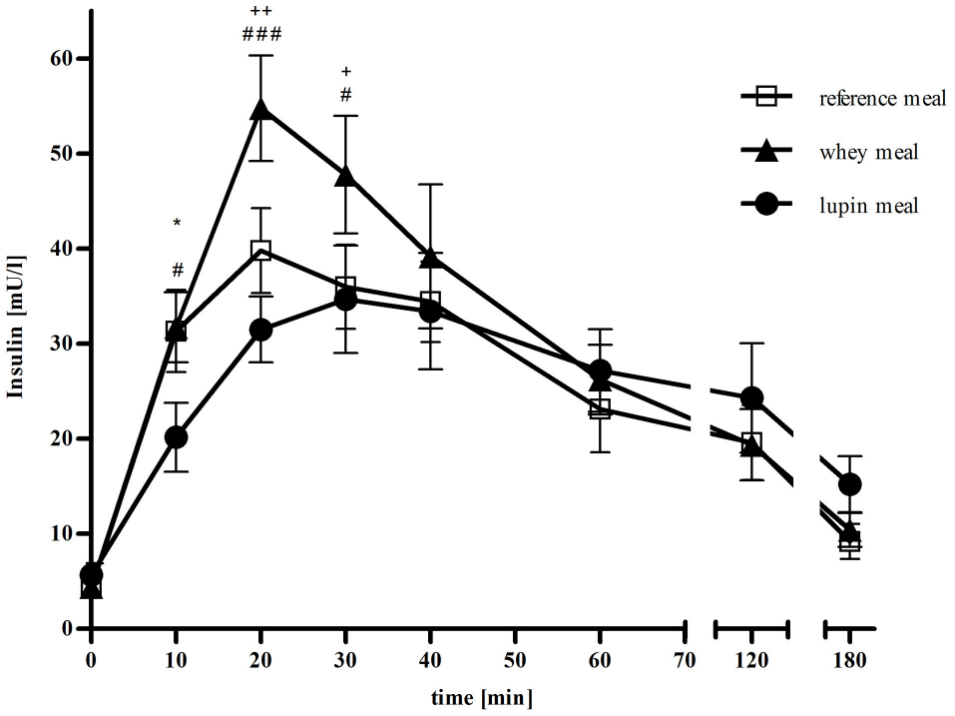
Mean (± SEM) of serum insulin concentrations in mU/l over 180 min following the three different meals: □ representing the reference meal, ▴ representing the meal supplemented with whey, • representing the meal supplemented with lupin, ^*^ representing differences between the lupin and the reference meal, + representing differences between the whey and the reference meal and # representing differences between the lupin and the whey meal. ^***/+++/###^*p* < 0.001; ^**/++/##^*p* = 0.001–0.01; ^*/+/#^*p* > 0.01–0.05.

Postprandial serum insulin concentrations are shown in Figure 3. All fasting serum insulin concentrations were within the normal range (reference: 4.27 mU/l; whey: 4.39 mU/l; lupin: 5.69 mU/l). Comparing the insulin response following the whey and the lupin meal, supplementation with lupin led to significantly lower serum insulin concentrations than supplementation with whey protein at time points +10 min (*p* = 0.037), +20 min (*p* < 0.001) and +30 min (*p* = 0.014). From time point +40 min onwards, no significant difference were observed (all *p* > 0.6). Supplementation with whey protein led to higher insulin responses at 20 and at 30 min after consumption when compared with the reference meal (*p* = 0.004 and *p* = 0.032, respectively. No such differences were observed at time points +10 min, and at +40 min to +180 min (all *p* > 0.90). Serum insulin concentrations differed between after lupin supplementation and after the reference meal at time point +10 min (*p* = 0.047)only, but not at any other time point (all *p* > 0.21).

## Discussion

In the present study, we have compared the effects of whey and lupin supplementation on postprandial blood glucose concentrations and the serum insulin response in healthy human participants in comparison to a reference, carbohydrate-rich, meal. Within the first 60 min, whey and lupin supplementation led to a comparable blunting of postprandial glycemia (whey: −46%; lupin: −54%, 0–60 min AUC, respectively). Whilst this may not be surprising, it is astounding that the insulin responses were quite different after the two meals. In addition, we find it remarkable that, despite the increased caloric load in the supplemented meals, the two supplementations did entail blunted hyperglycemic responses.

The observed glycemia-lowering effect of whey protein is consistent with previous studies. Pal et Ellis, for instance observed a lower incremental AUC for blood glucose concentrations when comparing whey to a turkey or egg albumin liquid test meal in healthy men (Pal and Ellis, 2011). A blunted hyperglycemic response by approximately 20% has also been shown in T2DM patients (Frid et al., 2005), and by more than 50% in healthy volunteers (Nilsson et al., 2004). By contrast, the insulin response in our study was seemingly higher at some postprandial time points, albeit was not when expressed as AUC. This finding is partly at odds with previous studies that have demonstrated a significant increase in the incremental insulin also in AUC after whey protein supplementation (Nilsson et al., 2004; Frid et al., 2005; Pal and Ellis, 2011).

Despite the glycemia-lowering potential of lupin, there are only a few human studies to date available. Dove et al. observed a glucose-lowering effect in diabetic adults when supplementing a glucose containing beverage with lupin kernel flour (Dove et al., 2011). Another study investigating the effect of lupin kernel flour in healthy participants found a reduced incremental glucose AUC after consuming lupin kernel flour-enriched bread compared with consuming white bread (Lee et al., 2006). While the study by Dove et al. led to significant higher insulin and C-peptide concentrations, the study of Lee et al. induced a reduced incremental insulin AUC, which is more consistent with the present study.

Despite the similarities in the postprandial glucose lowering effect of the supplements used in the present study, they differ in the evoked insulin response. In line with previous studies, whey protein mostly leads to an increase in insulin concentrations, either observed as AUC or, as in our study, only at certain time points. Thus, while lupin can lead to an increase and also to a decrease in serum insulin concentrations, there seems to be a consistent effect in lowering postprandial glycemia. For both, whey and lupin, the exact mechanism lowering postprandial glycemia is not yet known. Regarding whey protein, several studies have shown that certain amino acids, especially branch-chained amino acids, seem to act as insulin secretagogues (Loon et al., 2000; Nilsson et al., 2007). Furthermore, we have to take into account the higher amount of calcium provided with the whey meal. As natural trigger of the pancreatic islets of Langerhans it might lead to an increased insulin secretion (Mears, 2004). Hall et al. found a significant increase in plasma amino acids after whey preload with highly significant increases in serum branched-chain amino acids. In addition, they observed a significant increase in incretin hormone concentrations: Glucagon-like peptide (GLP-1) and glucose-dependent insulinotropic polypeptide (GIP) were both significantly increased after whey preload (Hall et al., 2003), an observation that is consistent with other studies (Frid et al., 2005; Ma et al., 2009).

The additional stimulation of insulin secretion by the whey induced rise of certain amino acids and incretin hormones may accelerate glucose uptake into muscle cells through the insulin-dependent pathway of glucose transporter 4 (GLUT4). A supplementation with whey protein might indirectly intensify the activation of that pathway leading to a faster decrease in postprandial glycemia. However, in our study, we did not observe such pronounced increases in insulin concentrations that were reported in other studies, while postprandial glycemia was reduced to a similar extent (Nilsson et al., 2004; Frid et al., 2005; Pal and Ellis, 2011). This might be due to the fact that the aforementioned studies used more insulin response stimulating reference meals, such as pure glucose beverages or high-GI meals. In addition, some studies worked with T2DM patients in whom the effects might be different to the ones in healthy men. In addition, the quality and quantity of the given supplementation might differ between the studies, leading to variable pronounced effects.

The mechanism underlying the reduced increase in postprandial blood glucose concentrations may to be different between whey and lupin. Two different lupin components seem to be mainly responsible for the hypoglycemic effect. Baldeón et al. tested purified alkaloids of *lupinus mutabilis* for their hypoglycemic impact in people with type 2 diabetes. The authors showed a significant reduced postprandial increase in blood glucose concentrations over 90 min. Serum insulin concentrations were not affected (Baldeón et al., 2012). In contrast, Bertoglio et al. and Lovati et al. tested the glucose lowering effect of γ-conglutin, a small glycoprotein in lupins (Duranti et al., 2008; Bertoglio et al., 2011; Rosa Lovati et al., 2012). In 2011, Bertoglio et al. tested purified γ-conglutin in several dosages in rats and healthy humans and the authors observed a dose-dependent reduced increase in postprandial glycemia (Bertoglio et al., 2011). Administration of γ-conglutin for 21 days to hyperglycemic rats attenuated the rise in plasma glucose and serum insulin concentrations significantly when compared to glucose-fed rats (Rosa Lovati et al., 2012). The same study also showed that γ-conglutin supports the activity of insulin and metformin in glucose consumption of HepG2 cells (Rosa Lovati et al., 2012), whereas other studies have demonstrated the internalization and phosphorylation of γ-conglutin (Capraro et al., 2010), the insulin-binding (Magni et al., 2004) and the insulin-mimetic action (Terruzzi et al., 2011) of the glycoprotein *in vitro*. Collectively, these studies suggest the involvement of γ-conglutin in the hypoglycemic effect of lupin.

However, the exact mechanism still remains unclear and *in vivo* studies have to be performed to investigate the role of γ-conglutin in decreasing postprandial glycemia without a corresponding insulin response. Thus, it is currently unknown how γ-conglutin is processed upon administration, or even whether the protein reaches the insulin receptor fully intact. Furthermore, it is not clear whether other lupin components, such as *e.g*. alkaloids or fiber intensify the effect of γ-conglutin. In addition, whether an insulin-mimetic action is the only mechanism leading to a reduced postprandial glycemia or whether other reactions, such as a delayed resorption, or an inhibition of hepatic gluconeogenesis also play a role, will have to be investigated. Because of these unknowns, we decided to perform the present study with integral lupin flour containing all lupin components. However, using integral lupin flour results in a higher consumption of fat, which was shown to influence insulin secretion. Whether this was the triggering factor for the lower insulin secretion remains unclear, since it was shown that high-fat diets could lead to both, insulin resistance and impaired insulin secretion (Fu et al., 2009; Salimi et al., 2016). Further studies are necessary to identify the components which are responsible for the glucose lowering effect and whether these components are as effective as whey protein. Additionally, further studies should evaluate, whether the lower glucose response occurs due to the protein supplementation itself or whether the type of protein is the triggering this effect. Future studies should also investigate the absorption and localization of glucose upon lupin administration.

## Limitations

As a first approach to the short-term effects of a lupin and whey supplementation on the postprandial blood glucose response, this study and budgetary restrictions led to some compromises. For, example, two participants had BMIs in the overweight range. However, their HbA1c levels were within normal range, and we therefore trust that inclusion of their data into the statistical analysis is justifiable. Additionally, BMI values varied substantially between male and female participants, which may raise questions regarding to homogeneity of subjects. On the other hand, variation in these anthropometric values may speak in the favor of generalizability of the study. However, future studies should be performed with two separate gender groups or with a smaller variation in BMI values within the participant cohort in order to reduce sources of variation, and in order to thus better establish the physiological mechanisms involved in the glucose and insulin responses. Additionally, meals could be supplemented with other nutrients, such as fat or calcium in order to balance the meals and to enable a better comparability of the meals. The same applies for a supplementation of the reference meal with another protein.

## Conclusions

In conclusion, the present study has shown that supplementation with lupin can reduce postprandial glycemia in healthy human participants, and perhaps even more than supplementation with whey protein. Even though the exact mechanism of action still remains unclear and we only conducted as short- term effect study with healthy participants, these results suggest that lupin can potentially serve as a vegetable alternative for whey protein in the control and prevention of glycemia.

## Author contributions

PF, WB, and JR conceived the idea for the experiment. KS and PF were responsible for the design of the study. KS and AE conducted the study. KS, AE, and BJ analyzed data and performed statistical analysis, and interpreted the results jointly with PF and JR. KS and PF wrote the manuscript and JR critically reviewed it. PF and WB had primary responsibility for final content of the paper. All authors read and approved the final manuscript.

## Funding

KS received a Helmholtz Space Life Sciences Research School (SpaceLife) PhD scholarship. SpaceLife was funded in equal parts by the Helmholtz Association (grant no.: VH-KO-300) and the German Aerospace Center (DLR).

### Conflict of interest statement

The authors declare that the research was conducted in the absence of any commercial or financial relationships that could be construed as a potential conflict of interest. The reviewer ACL and handling Editor declared their shared affiliation, and the handling Editor states that the process nevertheless met the standards of a fair and objective review.

## Abbreviations

BL: Baseline
BW: Body weight
CH: Carbohydrate
F: Fat
GI: Glycemic index
GIP: Glucose-dependent insulinotropic polypeptide
GL: Glycemic load
GLM: General linear model
GLP-1: Glucagon-like pepide 1
GLUT4: Glucose transporter 4
H_2_O: Dihydrogen monoxide
kJ: Kilo joule
LME: Linear mixed effects
PCOS: Polycystic ovary syndrome
P: Protein
SD: Standard deviation
SEM: Standard error of the mean
T2DM: Type 2 diabetes mellitus.
