# Supplementary figures and images for: Short-Term Effects of Lupin vs. Whey Supplementation on Glucose and Insulin Responses to a Standardized Meal in a Randomized Cross-Over Trial
Source: Front Physiol. 2017 Apr 10;8:198. doi: 10.3389/fphys.2017.00198 (PMC5385353; doi:10.3389/fphys.2017.00198)

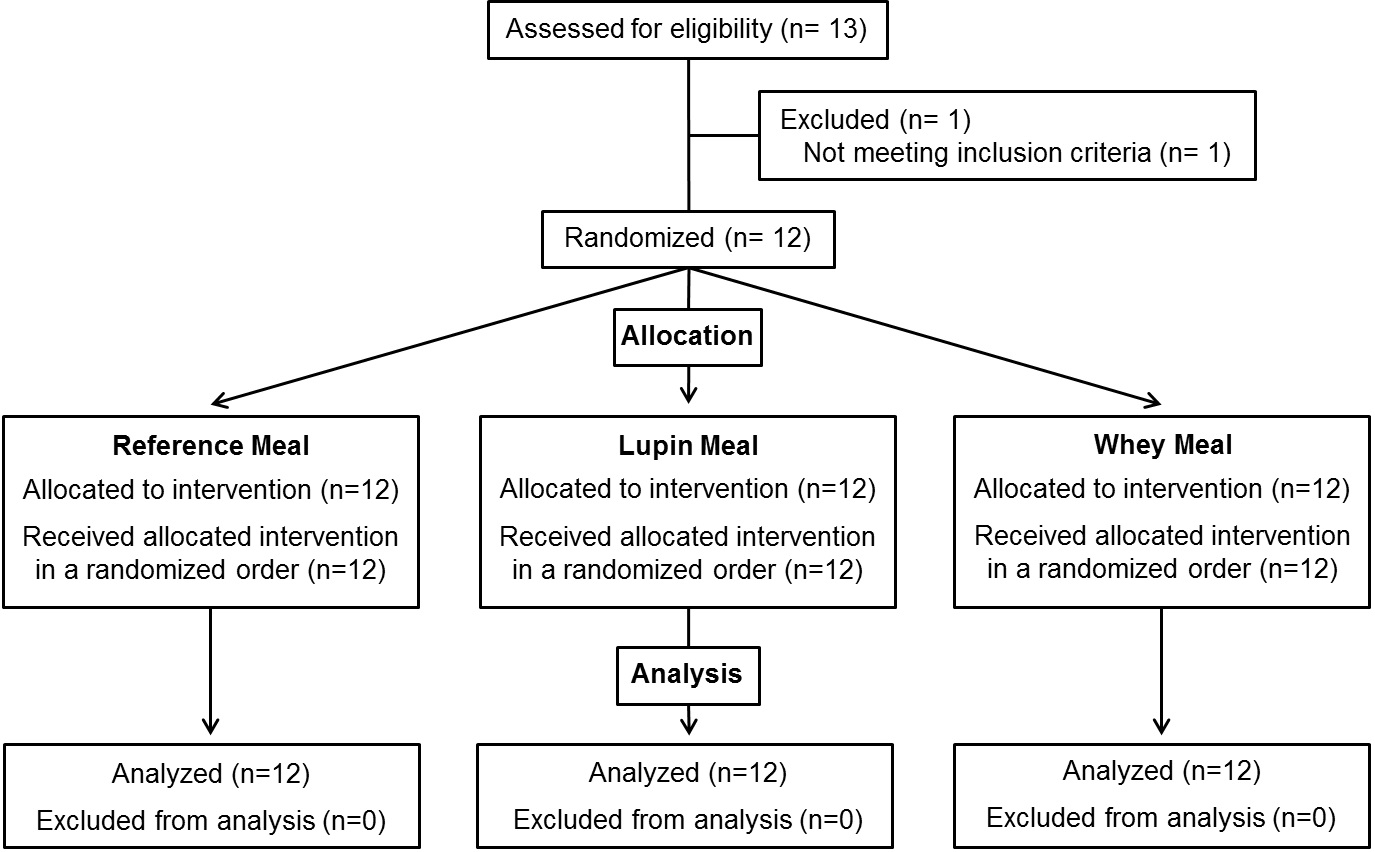

Supplement: Supplementary file 1 [file Image1.JPEG]
